# Self-compassion and professional quality of life among midwives and nurse assistants: A cross-sectional study

**DOI:** 10.18332/ejm/149520

**Published:** 2022-07-29

**Authors:** Karin Ängeby, Christine Rubertsson, Ingegerd Hildingsson, Malin Edqvist

**Affiliations:** 1 Women’s Department and Centre for Clinical Research Education, County Council of Varmland, Karlstad, Sweden; 2 Department of Health Sciences, School of Education, Health and Social Sciences, Dalarna University, Falun, Sweden; 3 Department of Health Sciences, Faculty of Medicine, Lund University, Lund, Sweden; 4 Department of Women's and Children's Health, Faculty of Medicine, Uppsala University, Uppsala, Sweden; 5 Clinical Epidemiology Division, Department of Medicine Solna, Karolinska Institutet, Stockholm, Sweden

**Keywords:** midwives, resilience, quality improvement, psychometric test, professional quality of life, self-compassion

## Abstract

**INTRODUCTION:**

Self-compassion and satisfaction derived from helping others is part of healthcare providers’ professional quality of life. The aim of this study was to explore and psychometrically test two instruments measuring self-compassion and professional quality of life among midwives and nurse assistants.

**METHODS:**

This was a cross-sectional study with midwives and nurse assistants working with intrapartum care at five different labor wards in Sweden. The Self-Compassion Scale (SCS) and the modified Professional Quality of Life Measurement (ProQOL) were validated and correlation analyses were calculated between the different subscales. Descriptive statistics, t-test, were calculated to analyze associations between the subscales of the SCS, the ProQOL and the background variables.

**RESULTS:**

Midwives were more self-critical than nurse assistants, and the midwives who were negative towards the new clinical practice scored higher for compassion fatigue. The principal component analysis showed a two-factor solution for both the SCS and the modified ProQOL. The two SCS subscales were named ‘self-criticism’ (α=0.85) and ‘self-kindness’ (α=0.87). The two ProQOL subscales were named ‘compassion satisfaction’ (α=0.83) and ‘compassion fatigue’ (α=0.78). A negative correlation was found between self-kindness and compassion fatigue subscales, between compassion satisfaction and compassion fatigue, and between self-kindness and self-criticism.

**CONCLUSIONS:**

The SCS and modified ProQOL are considered as valid questionnaires for use in a Swedish maternity setting and a correlation between the scales was found. Midwives are more self-critical than nurse assistants. Understanding and identifying compassion fatigue among midwives is important to managers responsible for quality improvement and practice changes.

## INTRODUCTION

Midwifery requires compassion and authenticity during labor and birth. When caring for women and their families, midwives need to deal with anxiety, pain, fear and sometimes grief as well as excitement and happiness^1^. Managing emotions in order to support women can leave midwives feeling emotionally drained^2^ and midwives are known to be at heightened risk of both burnout and secondary traumatic stress^3,4^. Being the second victim and experiencing secondary traumatic stress can contribute to a negative impact on the health provider’s professional quality of life including compassion fatigue and burnout^4,5^. One-third of midwives in a Swedish study reported symptoms of burnout, and one-third had experienced a work-related situation that made them consider leaving the profession. Lack of staff and a stressful work environment and age were the factors most strongly associated with burnout^6,7^. A recently published Swedish study found that midwives felt that they were not able to provide a safe and women-centered care due to the strained work situation^8^. How nurse assistants working in intrapartum care are affected is not known but research from other fields in nursing show that there were differences between registered nurses and nurse assistants in how they managed stressful situations^8^, and that nurse-assistants working in intensive care units were at higher risk for burnout than registered nurses^9^. A Swedish study exploring healthcare professionals’ perceptions of patient safety in connection to childbirth showed that midwives and nurse assistants worked closely together in order to avoid unnecessary interventions and support the birthing woman^10^.

The Swedish maternity care is free of charge and provided by midwives during labor and birth, and with midwives together with physicians if complications arise, where obstetricians take over the medical responsibility^11^. Midwives are assisted by nurse assistants, who are staff with a secondary level of education that support midwives with practical tasks and provide direct care to women during labor and birth^11,12^. All midwives in Sweden have a three-year nursing education with a Bachelor’s degree. The midwifery education is 1.5 years following the nursing education and comes with a one-year Master’s degree^13^.

To cope with the negative aspects of professional life, a level of resilience is needed. Resilience is when an individual, group or community responds more positively to an adverse situation than might be expected^1^. Self-care (i.e. taking care of our mental, emotional, and physical health) and the support of colleagues are considered to be crucial in creating resilience and in sustaining the joy and passion for midwifery practice. Being kind and understanding toward ourselves when encountering difficulties, shortcomings and failures is a form of self-care, also defined as self-compassion^14,15^. Neff^16^ proposes that self-compassion is a type of self-to-self relating that represents a compassionate rather than uncompassionate stance toward the self when faced with personal suffering: self-kindness versus self-judgment, a sense of common humanity versus isolation, and mindfulness versus overidentification^16^. Self-compassion has gained attention in recent years as a means of bolstering resiliency against stress, burnout, and emotional exhaustion^17^.

Even though the second stage of labor is an integral part of giving birth, it is acknowledged that this part of the process can be particularly demanding for midwives. In addition to giving emotional support, the midwife is responsible for the baby’s well-being and for preventing perineal trauma^18^. In Sweden, midwives are being helped by nurse assistants during the second stage of labor and only ask other midwives for assistance in complicated situations. In Sweden, there is an ongoing debate about prevention of severe perineal trauma. At the same time, there are few preventive strategies that are evidence-based, which makes practice decisions during the second stage challenging^18^. As all professions involved are aware of the risk of severe perineal trauma, there is a strong desire to do everything possible to prevent injuries, leading to new preventive strategies being implemented. Recently, a new clinical practice has been introduced in many Swedish hospitals to reduce severe perineal trauma. This procedure involves two midwives attending the woman during the second stage of labor in spontaneous vaginal births. This practice can be viewed as a complex health intervention and are evaluated in a multicenter randomized controlled trial (M-RCT)^19^. Professions involved in the trial are midwives assisting women giving birth spontaneously, together with nurse assistants who support the midwives and the women. The midwives will state in a case report form how they experienced having a colleague present. It could be hypothesized that having a colleague in the birthing room could be either a positive experience, but it might be challenging if the midwives feel that they are being observed and judged. This may further reinforce already experienced feelings of inadequacy and self-criticism. For the nurse assistant who are used to be the ones assisting the midwives, the new clinical practice may evoke questions regarding work tasks and their role in the birthing room. It is possible that they feel unimportant if two midwives assist the woman since their professional role in the birthing room is reduced. Therefore, the aim of the study was to explore and psychometrically test two instruments measuring self-compassion and professional quality of life among midwives and nurse assistants.

## METHODS

### Design

This is a cross-sectional study before the commencement of an M-RCT, using a survey to explore midwives’ and nurse assistants’ self-compassion and professional quality of life.

### Procedure and participants

Five labor wards had accepted to participate in a M-RCT, four University teaching hospitals and one County hospital. The study is described elsewhere^19^. All midwives and nurse assistants on these labor wards who attend births were scheduled to participate in a training session before the start of the M-RCT. The study was designed according to the principles of the Declaration of Helsinki. During these sessions, participants were given oral and written information regarding the study and consent to participate was obtained from 223 midwives and 76 nurse assistants that completed the questionnaire. Participation in the study was voluntarily and participants could withdraw from the study at any time, without affecting their relationship with managers or members of the research group. The researchers responsible for the training provided the survey and answered questions. The participants were given time to complete the survey during these sessions. They were instructed to answer the questions based on how they had felt in the past 30 days. The training sessions took place between 1 November 2018 and 4 December 2019, prior to each maternity ward entering the trial.

### Data collection

The Self-Compassion Scale (SCS) was used to measure self-compassion^16^. The SCS is designed to measure three main components of self-compassion on six separate subscales: self-kindness (5 items), common humanity (4 items) and mindfulness (4 items); and their opposites: self-judgment (5 items), isolation (4 items) and over-identification (4 items). Self-kindness is defined as extending kindness and understanding to oneself rather than harsh self-criticism and judgment. Common humanity means seeing one’s experiences as part of the larger human experience rather than as separating and isolating. Mindfulness is a process of openly attending, with awareness, one’s present moment experience^20^. The items in the SCS scale are rated on a 5-point Likert scale from 1 (almost never) to 5 (almost always). The SCS has been validated and the reported internal consistency for the total score is 0.92, while Cronbach’s alpha for the six subscales ranged from 0.80–0.88^16^. The SCS has been translated into Norwegian^21^ and several Swedish versions of the SCS exist, although to our knowledge none of the latter has been psychometrically tested. For this study we used a Swedish version of the scale that has been translated and back translated^22^. This version was compared to the validated scale from Norway^21^. The two translations were found to be similar, and the version translated by Häll^22^ was used without alterations.

The ProQOL was used to measure professional quality of life^23^. ProQOL measures compassion satisfaction (10 items) and compassion fatigue. Compassion fatigue is further divided into two parts: burnout (10 items) and secondary traumatic stress (10 items). Stamm^23^ describes compassion satisfaction as the satisfaction individuals derive from work related to caring for others. Compassion fatigue is the negative aspect of helping those who experience traumatic stress and suffering. Secondary traumatic stress and burnout are considered as two elements of compassion fatigue^23^. Burnout is defined as loss of energy, dissociation from work, depersonalization, and emotional exhaustion, all caused by stress^24^. The ProQOL scale uses a 5-point Likert scale from 1 (never) to 5 (always). The Swedish translation can be found on the ProQOL website^23^. Internal consistency is reported to vary between 0.78 and 0.82, depending on study sample^25^. Lately, there has been some debate about the reliability and validity of the ProQOL for the burnout and the secondary traumatic constructs^25^. After performing a Rasch analysis Heritage et al.^26^ suggest a 21-item solution rather than the current 30 items. Following their discussions, a modified version of the ProQOL was used with the first 20 items of the 30 in the original scale. All previously named subscales were covered by the 20 items used.

Background data collected from the participants were profession, years of clinical experience in maternity care and working hours (working dayshift or nightshift, or rotation all hours). Furthermore, the participants were asked whether they were positive or negative toward the clinical practice of two midwives assisting the woman during the active phase of the second stage of labor.

### Statistical analysis

Descriptive statistics for the 26 items in SCS and the 20 items in the Modified ProQOL were calculated. The data were checked for normality as assessed by histogram, box plots, and levels of skewness, and kurtosis. To psychometrically test the SCS and the ProQOL, principal component analyses were used following the three steps described by Pallant^27^. The two different instruments were analyzed separately. For both scales the suitability of the analysis was assessed by using Bartlett’s test of sphericity. The sample adequacy was tested with Kaiser-Meyer-Olkin (KMO). The two scales with 26 items and 20 items, respectively, were then subjected to extraction of factors. Principal component analysis was used to create components of all the variance rather than factors. Kaiser’s criterion was used for testing the eigenvalue of the factors, and factors with an eigenvalue >1.0 were obtained for further analysis. Coefficients <0.40 were suppressed, and the items were included in the component where they loaded the highest. Cattell’s scree test was used for a visual decision and, finally, a parallel analysis, which involves comparing the size of the eigenvalues with those obtained from a randomly generated data set of the same size, was performed^28^. In the final step, Oblimin rotation was used to explore the structure of all the items, since the factors might correlate. Participants with incomplete surveys were excluded in the principal component analysis but included in the descriptive subgroup analysis. Finally, a reliability test for the total scales showed a Cronbach alpha value of 0.67 for the SCS and 0.64 for the ProQOL, and a value >0.70 was regarded as desirable. Construct validity, including divergent and convergent validity, were assessed through bivariate Pearson’s correlations among the SCS and the modified ProQOL mean scores. Effect size was interpreted according to Cohen’s d; small effect 0.2, 0.5 moderate effect, and 0.8 large effect^29^. Descriptive statistics were calculated as means, standard deviations (SD) and percentages. To analyze possible associations between the subscales of the SCS, the ProQOL and the background variables (profession, work experience, working hours), independent samples t-tests were calculated with statistical significance level set at <0.05.

## RESULTS

### Background characteristics of participants

A total of 223 midwives and 76 nurse assistants completed the questionnaire, corresponding to a response rate of 96%, since only ten of those who were offered to complete the survey declined. The majority of the participants were midwives (Table 1).

**Table 1:** Overview over the participants (N=309)

*Characteristics*	*n (%)*
**Profession**	
Midwife	223 (72.2)
Assistant nurse	76 (24.6)
Missing	10 (3.2)
**Shift work**	
Day and evening	137 (44.3)
Nightshift only	78 (25.2)
Three-shift	84 (27.2)
Missing	10 (3.2)
**Working experience in labor ward** (years)	
<1	43 (3.9)
1–2	37 (12)
2–5	66 (21.4)
6–10	38 (12.3)
11–20	49 (15.9)
>20	67 (21.7)
Missing	9 (2.9)
**Midwives’ attitudes toward the new clinical practice**	
Very positive	183 (59.2)
Partly positive	81 (26.2)
Neutral (not positive or negative)	24 (7.8)
Negative	8 (2.6)

Almost half of the participants (44%) worked day and evening shifts, 25% only night shifts and 27% all three shifts. Similarly, almost half of the participants had <10 years’ work experience and half of them >10 years. The vast majority of the participants reported a positive attitude toward the new clinical practice involving two midwives during the second stage of labor. Only a few (10%) reported a neutral or negative attitude toward the intervention.

### Psychometric test

Table 2 presents the psychometric test for the Self-Compassion Scale and the modified version of the Professional Quality of Life Measure for the total sample of midwives and nurse assistants.

**Table 2:** Pattern Matrix of two components-solution from principal components analysis with Oblimin rotation

*Self-Compassion Scale*		*Mean (SD)*	*Median (IQR)*	*Range*	*Cronbach alpha*
**Self-criticism** (n=293)	Items=13	36.04 (10.17)	36 (29.5–43)	13–64	0.85
When I see aspects of myself that I don’t like, I get down on myself.	0.771				
When times are really difficult, I tend to be tough on myself.	0.751				
When I fail at something important to me, I become consumed by feelings of inadequacy.	0.747				
When I’m feeling down, I tend to obsess and fixate on everything that’s wrong.	0.744				
When I think about my inadequacies, it tends to make me feel more separate and cut-off from the rest of the world.	0.739				
When I fail at something that’s important to me, I tend to feel alone in my failure.	0.735				
I can be a bit cold-hearted towards myself when I’m experiencing suffering.	0.712				
I’m disapproving and judgmental about my own flaws and inadequacies.	0.7				
When I’m really struggling, I tend to feel like other people must be having an easier time of it.	0.683				
I’m intolerant and impatient towards those aspects of my personality I don’t like.	0.659				
When I’m feeling down, I tend to feel like most other people are probably happier than I am.	0.652				
When something painful happens, I tend to blow the incident out of proportion.	0.601				
When something upsets me, I get carried away with my feelings.	0.437				
**Self-kindness** (n=287)	Items=12	33.68 (6.21)	33 (30–37)	13–50	0.87
When things are going badly for me, I see the difficulties as part of life that everyone goes through.	0.725				
When I’m down and out, I remind myself that there are lots of other people in the world feeling like I am.	0.659				
When something painful happens, I try to take a balanced view of the situation.	0.652				
When I fail at something important to me, I try to keep things in perspective.	0.632				
I try to see my failings as part of the human condition.	0.618				
I try to be loving towards myself when I’m feeling emotional pain.	0.609				
I try to be understanding and patient towards those aspects of my personality I don’t like.	0.573				
When I feel inadequate in some way, I try to remind myself that feelings of inadequacy are shared by most people.	0.558				
When I’m going through a very hard time, I give myself the caring and tenderness I need.	0.533				
I’m tolerant of my own flaws and inadequacies.	0.488				
When I'm feeling down, I try to approach my feelings with curiosity and openness.	0.448				
I’m kind to myself when I’m experiencing suffering.	0.439				
Not loading					
When something upsets me, I try to keep my emotions in balance.					
**Modified professional quality of life**					
Compassion satisfaction (n=291)	Items=10	41.67 (4.61)	42 (39–45)	26–50	0.83
My work makes me feel satisfied.	0.721				
I like my work as a [helper].	0.714				
I feel invigorated after working with those I [help].	0.673				
I have happy thoughts and feelings about those I [help] and how I could help them.	0.657				
I am the person I always wanted to be.	0.638				
I am happy	0.631				
I feel connected to others.	0.536				
I am pleased with how I am able to keep up with [helping] techniques and protocols.	0.513				
I have beliefs that sustain me.	0.421				
**Compassion fatigue** (n=290)	Items=8	15.72 (4.56)	15 (12–19)	8–29	0.78
I think that I might have been affected by the traumatic stress of those I [help].	0.775				
I feel depressed because of the traumatic experiences of the people I [help].	0.723				
I am not as productive at work because I am losing sleep over traumatic experiences of a person I [help].	0.704				
I feel as though I am experiencing the trauma of someone I have [helped].	0.694				
I feel trapped by my job as a [helper].	0.549				
I find it difficult to separate my personal life from my life as a [helper].	0.547				
I jump or am startled by unexpected sounds.	0.539				
Because of my [helping], I have felt ‘on edge’ about various things.	0.458				
**Not loading**					
I am preoccupied with more than one person I [help].					
I feel worn out because of my work as a [helper].					

Analysis with 2-factor solution from Principal Component Analysis with Oblimin rotation. Only major factor loadings and loadings above 0.40 are displayed.

### Self-Compassion Scale (SCS)

The entire survey for Self-Compassion Scale was completed by 287 participants. The KMO value was 0.92, and Bartlett’s test of sphericity (p<0.001) was statistically significant. The principal component analysis showed a two-factor solution. The pattern showed items loading between 0.44 and 0.77. One item (‘When something upsets me, I try to keep my emotions in balance’) loaded below 0.30. After removing this item and conducting factor analysis on the remaining 24 loading items, 45.6% of the variance was explained. The original SCS scale has a six-factor solution, and a total self-compassion score can be calculated. However, in this sample, the PCA analysis of the Self-Compassion Scale revealed a two-component solution, and the two subscales were named ‘self-criticism’ and ‘self-kindness’. The self-criticism subscale contained 13 items, with score ranging 13–64, with a mean of 36.04 (SD: 10.17) and a Cronbach alpha of 0.85. The self-kindness subscale, contained 12 items, ranging 13–50, with mean of 33.68 (SD: 6.21) and a Cronbach alpha of 0.87.

### Professional Quality of Life – modified version

The sample consisted of 290 participants who completed the entire questionnaire for ProQOL. The KMO value was 0.85 and Bartlett’s test of sphericity was statistically significant (p<0.001). The principal component analysis showed a two-factor solution, with 18 items explaining 38.4% of the variance. All the included items had factor loadings between 0.42 and 0.78. Two items had low loadings (<0.30) and are presented as single items: ‘I am preoccupied with more than one person I help’ and ‘I feel worn out because of my work as a helper’. When the factor analysis was conducted on the remaining 18 loading items, this solution explained 41.4% of the variance. The modified ProQOL contained two subscales that were named ‘compassion satisfaction’ and ‘compassion fatigue’. The items that were added to each scale were either positively expressed and fitted in the subscale compassion satisfaction, or negatively expressed and corresponded to the subscale compassion fatigue, and consequently no items needed to be reversed. The subscale compassion satisfaction contained 10 items, ranging 26–50, mean of 41.67 (SD: 4.61) with a Cronbach alpha of 0.83. The other subscale named compassion fatigue, comprised 8 items, ranging 8–29, mean of 15.72 (SD: 4.56) and Cronbach alfa of 0.78.

### Correlational analysis between SCS and the modified ProQOL subscales

Pearson’s coefficient correlations for all four subscales are presented in Table 3. All correlations were statistically significant. A negative correlation was found between self-kindness and compassion fatigue (r= -0.233, n= 278, p<0.01). As expected, a negative correlation between compassion satisfaction and compassion fatigue was found (r= -0.314, n=285, p <0.001). Furthermore, compassion satisfaction was also significant and inversely related to self-criticism (r= -0.352, n=284, p<0.001). Another expected finding was the negative correlation between self-kindness and self-criticism (r= -0.465, n=283, p <0.001).

**Table 3:** Correlations between the SCS and the modified ProQOL subscales

		*Self-criticism (SCS)*	*Self-kindness (SCS)*	*Compassion satisfaction (mod. ProQOL)*	*Compassion fatigue (mod. ProQOL)*
Self-criticism (SCS)	Pearson correlation	1	-0.465**	-0.352**	0.415**
	Sig. (2-tailed)		0	0	0
	N	293	283	284	283
Self-kindness (SCS)	Pearson correlation	-0.465**	1	0.355**	-0.233**
	Sig. (2-tailed)	0		0	0
	N	283	287	278	278
Compassion satisfaction (mod. ProQOL)	Pearson correlation	-0.352**	0.355**	1	-0.314**
	Sig. (2-tailed)	0	0		0
	N	284	278	291	285
Compassion fatigue (mod. ProQOL)	Pearson correlation	0.415**	-0.233**	-0.0314**	1
	Sig. (2-tailed)	0	0	0	
	N	283	278	285	290

** Correlation is significant at the 0.01 level (2-tailed).

### Relationship between background characteristics, the SCS and the modified ProQOL

Table 4 shows the relationship between background characteristics and the subscales of the SCS and the modified ProQOL.

**Table 4:** Self-Compassion and modified ProfQoL regarding intercorrelations, reliability and descriptive statistics

	*Self-Compassion Scale*		*Modified Professional Quality of Life Scale*	
	*Self-criticism (n=293)*	*Self-kindness (n=287)*	*Compassion satisfaction (n=291)*	*Compassion fatigue (n=290)*
**Comparisons between subscalescores and professions**, mean (SD)				
Midwives (n=218)	37.19 (9.65)	33.93 (6.24)	41.55 (4.55)	15.73 (4.44)
Assistant nurses (n=75)	32.76 (11.01)	32.90 (6.12)	42.05 (4.85)	15.65 (4.91)
t-test	t=3.08, df=113.46, **p=0.003**	t=1.20, df=284, p=0.23	t=-0.81, df=287, p=0.42	t=0.13, df=287, p=0.90
Effect size	**0.45**	0.17	0.11	0.02
**Comparisons between subscalescores and years of profession for unexperienced midwives (<2 years) and those with experience (>2 years)**, mean (SD)				
<2 (n=79)	36.10 (11.77)	33.56 (6.07)	42.27 (4.59)	14.44 (4.29)
>2 (n=211)	36.04 (9.58)	33.67 (6.24)	41.41 (4.60)	15.84 (4.66)
t-test	t=0.05, df=290, p=0.97	t=-0.13, df=284, p=0.90	t=-1.41, df=288, p=0.16	t=-0.67, df=287, p=0.51
Effect size	0.01	0.02	0.20	0.31
**Comparisons between subscalescores and years of profession for very experienced midwives (>20 years) and those with experience (<20 years)**, mean (SD)				
>20 (n=64)	33.94 (8.60)	33.61 (5.90)	41.00 (4.70)	15.76 (4.26)
<20 (n=228)	36.65 (10.53)	33.65 (6.27)	41.83 (4.57)	15.72 (4.65)
t-test	t=-1.89, df=290, p=0.06	t=-0,052, df=284, p 0.958	t=-1.27, df=288, p=0.21	t=-0.05, df=287, p=0.96
Effect size	0.28	0.01	0.18	0.09
**Comparisons between subscalescores and positive attitude and neutral/negative towards the new clinical guideline, mean** (SD)				
Negative (n=32)	35.94 (8.73)	32.72 (5.63)	41.58 (3.85)	17.47 (5.27)
Positive (n=255)	35.99 (10.38)	33.83 (6.27)	41.74 (5.23)	15.4 (4.32)
t-test	t=-0.03, df=287, p=0.98	t=-0.916, df=281, p=0.37	t=-0.18, df=284, p=0.858	t=2.138, df=36.48, **p=0.039**
Effect size	0.01	0.19	0.03	**0.43**

When comparing the two subscales and profession, a statistically significant difference was found for the subscale self-criticism, where the midwives scored higher than the nurse assistants (p=0.003) with a small effect size (Cohen’s d=0.40). For the subscale self-kindness, no statistically significant differences were found between the two professions.

As self-criticism was not associated with work experience among midwives, a refined analysis was undertaken to explore whether inexperienced (<2 years), or very experienced (>20 years), midwives were more self-critical or self-kind. The results of this analysis did not alter the findings and showed that inexperienced midwives did not score significantly higher than more experienced ones, for the subscales self-criticism and self-kindness. However, the very experienced midwives (>20 years) scored lower for self-criticism than the more inexperienced, a difference that did not reach statistical significance (p=0.06). No associations were found in being very inexperienced or very experienced and self-kindness (Table 4).

For the modified ProQOL, there were no statistically significant differences regarding profession and the two subscales. As for the SCS subscales, there were no statistically significant differences when comparing the least experienced (<2 years), with more experienced, midwives and vice versa. However, midwives who were neutral or negative toward the practice scored significantly higher for compassion fatigue, compared to those who were positive (p=0.04), with a small effect-size (Cohen’s d=0.40).

## DISCUSSION

Our results show that the SCS and modified ProQOL are valid scales to use in the Swedish maternity setting. Midwives were more self-critical than nurse assistants. In addition, those who scored higher for compassion fatigue also scored higher in self-criticism and lower in self-kindness. Furthermore, the midwives who scored higher for compassion fatigue were more negative toward the new practice to reduce perineal trauma.

To our knowledge there is little research regarding self-criticism and self-compassion among midwives and nurse assistants. However, the findings of the present study are in line with those of Juthberg et. al.^8^, where occupational belonging explained a variance in stress of conscience. Katsantoni et al.^4^ found a strong correlation between burnout and compassion fatigue, and the professionals who had experienced a traumatic birth experience reported a significantly higher level of compassion fatigue and secondary traumatic stress^4^. Even if self-criticism is known to be a vulnerability personality trait for the development of psychosocial impairment^30,31^, it is also associated with correcting or improving oneself^32^. The difference between the professions might be explained by the fact that midwives are the primary caregivers for laboring women and their roles differ in responsibility and accountability. In contrast to nurse assistants, they have a professional responsibility and will be held accountable if an adverse event occurs. Wahlberg et al.^33^ describes the vulnerability, insecurity, and feelings of loneliness among midwives after a severe event. Given this, midwives may be more self-critical in their judgements of self-performance in attempts to improve standards and/or prevent errors.

The midwives and nurse assistants who experience compassion fatigue judged themselves more harshly and were less kind and understanding toward themselves. This result is in line with the findings of Beaumont et al.^34^, where the student midwives who scored higher in self-judgment subscale also reported greater compassion fatigue and with the study from Katsantoni et al.^4^. If those who provide care to women in labor ‘perform’ feelings rather than provide authentic support, this can decrease their emotional engagement and even lead to cynicism^2,35^. Midwives who score higher for compassion fatigue and lower in self-compassion may be at risk of developing client-related burnout. Client-related burnout comprises feelings of not being able to offer any more of oneself at work^4,35^. It can be argued that this type of exhaustion among professionals has the most serious consequences for women, since providers suffering from compassion fatigue are not able to provide compassionate and authentic care. Therefore, it is interesting to note that being negative or indifferent towards the new clinical practice to prevent severe perineal trauma was associated with compassion fatigue. Deery et al.^2^ consider that this type of emotional draining does not only have consequences for women but also undermines the potential for excellent practice. According to Nilsen et al.^36^ practice changes are more difficult to achieve when the professionals feel resigned and tired. Compassion fatigue among midwives and nurse assistants is therefore an issue that is important for managers to address. Firstly, regarding the women in labor, who might be affected, and secondly regarding organizations which are obliged to work actively with quality improvement and implementation. The work culture also affects the professional’s professional quality of life. Since different maternity wards have different rules about how team members are expected to cope with difficulties and emotions, a compassionate organizational culture with clinical supervision and an ongoing education can bolster and protect against compassion fatigue and burnout^17^.

According to the COSMIN checklist for evaluating the measurement properties of a scale, four key aspects of a scale require consideration and should be assessed when using a survey in a new setting: reliability, validity, responsiveness, and interpretability^37^. When using a survey in a new setting, a psychometric test is therefore suggested. The SCS has been used to assess self-compassion among midwifery students^34^ but to our knowledge, its psychometric properties have not been evaluated with midwives and nurse assistants working with intrapartum care. The present study resulted in a two-factor solution for SCS representing one positive dimension (self-kindness), and a negative dimension containing items related to self-criticism, instead of the six-factor solution. The original scale has been validated in different populations with mixed results regarding the six-factor solution^38^ and the two-factor solution in this study is identical with the findings from López et al.^38^ and Costa et al.^39^. There is a current discussion about whether self-compassion should be measured as a global construct, or whether it is better measured as two separate constructs. Our result reinforces the argument of Gilbert et al.^32^ who suggest that self-compassion is distinct from self-criticism, related to different affective and physiological systems, and therefore they should not be measured as one.

The ProQOL measurement was used to measure compassion satisfaction and compassion fatigue among midwives and nurse assistants. As suggested by Heritage et al.^26^, fewer of the items were used. The psychometric test revealed a two-factor solution, compassion satisfaction and compassion fatigue, and the original three factor solution with compassion satisfaction, secondary traumatic stress and burnout, could not be established. This finding is in line with previous research^26,40^. Heritage et al.^26^ argue that compassion fatigue can be meaningfully created as one construct to examine the broader negative facets of healthcare provision, and their anticipated outcomes. Geoffrion et al.^41^ suggest that compassion satisfaction and compassion fatigue represent higher and lower levels of the same construct rather than two different ones.

### Strengths and limitations

There are a number of limitations in this study. First, the only background variables collected were working experience and shift work. Personal information would have provided richer information and could have contributed to the interpretation of the findings as well as information about work related characteristics such as personal problems and traumatic work experience for midwives and nurse assistants. Such events can affect the results. Another possibility could have been exploring the relationship between organizational management and job satisfaction^42^. The decision to use and modify the ProQOL might be questioned in hindsight, as the scale has not been culturally adapted and as the subscale of burnout could not be validated. A preferable alternative might thus have been to add a scale that only measures burnout. However, the survey had become even more extensive and time consuming, something that was desirable to avoid.

The strengths of the study are the high response rate and the geographical spread of the participating labor wards, which increases the generalizability of the findings. The participating clinics account for approximately 18% of all births annually in Sweden. In addition, all the participants were given sufficient time and the same instructions during the structured training sessions. Further, they had the opportunity to have potential questions clarified by the researchers.

## CONCLUSIONS

This study showed that midwives are more self-critical than nurse assistants. Understanding and identifying compassion fatigue among midwives is of importance for managers that want midwives to provide high-quality care and work with quality improvement and practice changes. The SCS and the modified version of the ProQOL are valid instruments to use in Swedish maternity settings and can be used as tools to identify the need of organizational support, in settings where midwives have a high workload and where there is a risk for client related burnout. Further research should focus on interventions to increase midwives’ self-compassion and to reduce excessive self-criticism.

## Data Availability

The data supporting this research are available from the authors on reasonable request.
